# Mechanisms of blunt liver trauma patterns: An analysis of 53 cases

**DOI:** 10.3892/etm.2012.837

**Published:** 2012-11-27

**Authors:** WANGXUN JIN, LIMING DENG, HEPING LV, QIYU ZHANG, JINYING ZHU

**Affiliations:** 1 Department of General Surgery, The First Affiliated Hospital of Wenzhou Medical College, Wenzhou 325000;; 2 Department of Abdominal Tumor Surgery, Zhejiang Provincial Cancer Hospital, Hangzhou 310000;; 3 Department of Radiology, The First Affiliated Hospital of Wenzhou Medical College, Wenzhou 325000, P.R. China

**Keywords:** liver trauma, mechanism, blunt force

## Abstract

Blunt liver trauma is the most dangerous and the second most frequent solid organ trauma that occurs in the abdominal cavity. Management of this life-threatening situation remains a significant challenge. The present study identified that the patterns of blunt liver trauma were closely correlated with the characteristics of the blunt force. Illustrations of findings from this study have been included in the hope that they may aid surgeons in improving the management of this emergency. In total, 53 cases of blunt liver trauma that underwent laparotomy in the First Affiliated Hospital of Wenzhou Medical College between 1999 and 2009 were retrospectively studied. The cause of the injury, the direction and site of the blunt force, surgical records and CT films were carefully studied to obtain information on the patterns and severity of the liver injury and the correlation with blunt forces. Trauma in the right lobe of the liver was mainly caused by acceleration, deceleration and compression of the liver, while in the left lobe of the liver, acceleration was the main cause of the trauma. Liver lacerations were always located close to the attachment sites of the ligaments which bore the majority of the shearing stress. The characteristics of the blunt force play a key role in the different patterns of blunt liver trauma. A thorough understanding of the mechanisms of blunt liver trauma may aid doctors in the management of patients with this condition.

## Introduction

The liver is the second most frequently injured solid organ in the abdominal cavity. Blunt liver trauma demands emergency treatment and remains a great challenge for surgeons (1,2). Normally, the rib cage and the vertebral column protect the liver from violent forces from outside the body; the ligaments and vena cava fix the liver in place to avoid violent movements (3). However, under certain extreme circumstances, the rib cage and the ligaments take part in the liver trauma. There are two previously described mechanisms of blunt liver trauma, deceleration injury and crush injury (1,2,4). The patterns of blunt liver trauma are closely correlated with the characteristics of the blunt force. The direction, velocity, grade and site where the blunt force lands, as well as the position and motion of the victim when the injury occurred, all contribute to the patterns and severity of the blunt liver injury. The present study utilizes illustrations to demonstrate the patterns of blunt liver trauma following various blunt forces, in the hope that in this way the mechanisms of blunt liver injury may be clearly understood and memorized, therefore aiding emergency surgeons to improve the management of major blunt liver injuries.

## Patients and methods

A total of 53 blunt liver trauma patients who underwent surgery in the First Affiliated Hospital of Wenzhou Medical College between 1999 and 2009 were included in this study. The doctor’s and nurse’s notes were reviewed for information on gender, age and hospital stay. Special attention was given to the cause of the injury, the direction of the blunt force and the site on which it landed. Surgical records and CT films were particularly studied to obtain information on the patterns and severity of the liver injuries. Overall, 47 male and 6 female patients aged between 15 and 71 years old (mean, 37.5 years) were included in this study. All CT scans were performed and reviewed by qualified doctors. Experienced surgeons performed all surgical procedures and the details of these procedures were recorded carefully. The study was approved by the ethics committee of the First Affiliated Hospital of Wenzhou Medical College, Wenzhou, China. Written informed patient consent was obtained from the patient’s family.

## Results

### 

#### General items

At the time of admission, the mean blood pressure of the 53 patients was 12.4±4.6/7.4±3.3 kPa and the mean heart rate was 103±19 beats per min. The mean hospital stay was 16±10 days. Multiple injuries and organ dysfunctions resulted in a mortality rate of ∼19% (10 patients). In total, 44 patients had multiple injuries. Of these, 25 patients had coexisting thoracic injuries, the majority in the form of multiple rib fractures and lung contusions. Pelvic bone fractures and bladder ruptures were diagnosed in 3 patients, while 2 patients had splenic injuries, 1 patient had a right renal laceration and 2 patients suffered spine bone fractures. The average intraperitoneal hemorrhage volume was 2,144±1,469 ml and the mean quantity of red blood cells infused was 10 units. A CT was not used in 22 patients.

#### Characteristics of the blunt force

Injuries were caused by traffic accidents in 35 patients and by falls from varying heights in 10 patients, while the remainder were injured by being hit with fists (3 patients) and heavy objects (5 patients). A total of 30 patients had records on the site of the blunt force; the majority landed on the upper right quadrant or the belly. ‘Hit’ or ‘crush’ were the words most frequently used to depict the way in which the blunt force occurred. No details were provided as to the velocity of the blunt force or the motion of the victims in the accidents.

#### Scale and patterning of the blunt liver trauma

According to the American Association for the Surgery of Trauma (AAST) liver injury scale (1994 version) (5), every trauma was grade II or beyond; there were 5 grade II, 8 grade III, 27 grade IV and 13 grade V. During surgery, 32 patients suffered active bleeding. To control this bleeding, 5 patients received hepatic artery ligation, while the remaining patients underwent either direct ligation of the bleeding or suturing of the lacerated liver. A total of 36 traumas (67.9%) occurred in the right lobe of the liver, 18 of which had lacerations between the anterior and posterior lobes. Liver lacerations along Cantlie’s line were identified in 7 patients. A total of 9 traumas (17%) were in the left lobe of the liver and of these, 7 patients had a laceration either to the left or to the right of the falciform ligament. Only 1 patient (1.9%) suffered trauma in Couinaud segment I. With regard to injuries to the right lobe of the liver, the majority were irregular in shape (stellate).

## Discussion

### 

#### Mechanisms of blunt liver trauma

There were previously two well-recognized mechanisms of blunt liver trauma: deceleration injury and crush injury (1,2,4). In the present study, acceleration injury has been identified as another mechanism. The following descriptions and illustrations aim to demonstrate the mechanisms of blunt liver trauma clearly and accurately.

### Acceleration injury

#### Acceleration injury in the right lobe of the liver

**Figure 1.**
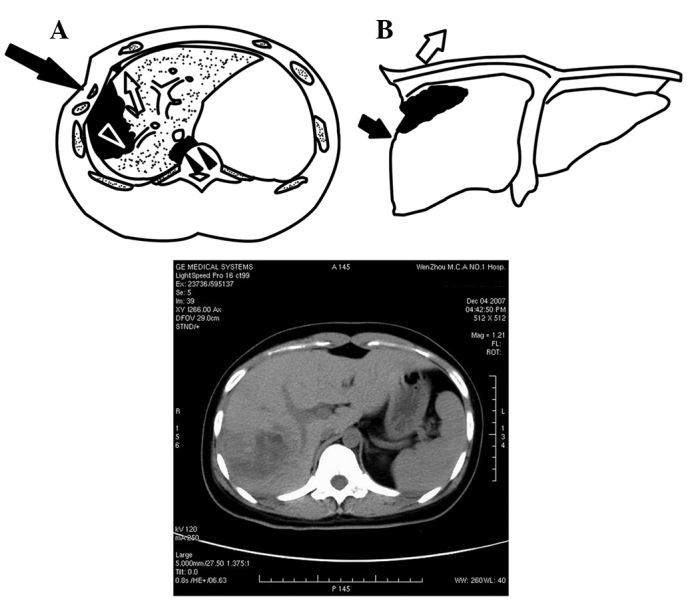
Acceleration injury in the right lobe of the liver caused by blunt forces from a lateral right direction. The right triangular ligament attaches to the site between Couinaud segments VII and VIII, making segment VII relatively fixed [(A and B) empty arrow] while the anterior lobe continues to move violently [(A) empty arrowhead and (B) black arrow). The black arrow in (A) indicates the blunt force. CT film shows a hepatic injury caused in this way by a traffic accident.

Although the right lobe of the liver is under the protection of the rib cage, it is susceptible to blunt forces when the speed or impact are large enough. Trauma in the right lobe accounted for 67.9% of all traumas in the present study, which is consistent with previous studies (1,2,6). These types of injuries are usually caused by forces from a lateral, right direction. The force lands on the chest wall, depresses it and then swiftly propels the liver. The right triangular ligament attaches to the site between Couinaud segments VII and VIII, making segment VII relatively fixed while segments VIII and V continue to move violently. This explains why lacerations are most frequently located between the anterior and posterior lobes. In the present study, out of the 36 right lobe lacerations, 18 cases were of this category. Under huge forces and high speeds, these lacerations may occasionally reach the inferior vena cava where the hepatic veins join, and result in the laceration of the retrohepatic vena cava and major hepatic vein (Fig. 1) (4,7–10).

**Figure 2.**
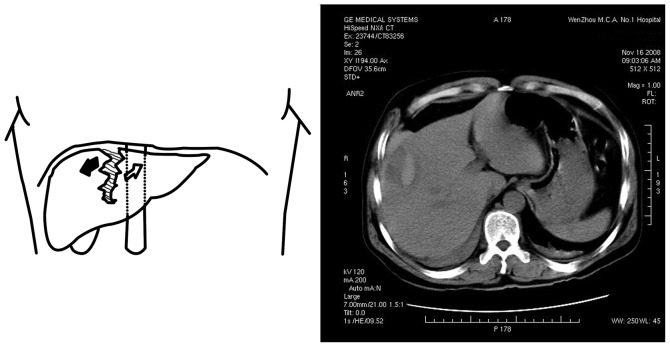
When the liver is propelled in an anterior-posterior direction, the retrohepatic vena cava fixes the liver (empty arrow), and the liver lacerates along Cantlie’s line due to the acceleration of the right lobe of the liver (black arrow). CT film shows a hepatic injury caused in this way by a traffic accident.

When the blunt force comes from the front, the liver is propelled in an anterior-posterior direction. As the vena cava is relatively fixed and serves as a counterforce when the right lobe is pushed towards the back, the liver lacerates along Cantlie’s line. The existence of the plane along Cantlie’s line also makes this site weaker and less able to resist the force causing the laceration (Fig. 2) (3–5,11).

#### Acceleration injury in the left lobe of the liver

**Figure 3.**
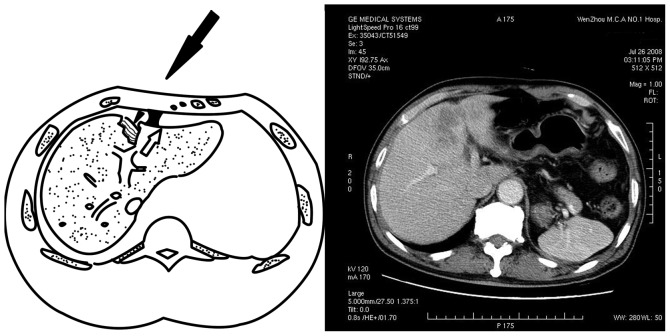
Acceleration injury in the left lobe of the liver. When the blunt force (black arrowhead) pushes the left lobe of the liver toward the back, the falciform ligament serves as a counterforce (empty arrow). The section of the liver that bears the shearing stress moves posteriorly and is lacerated. CT film shows a hepatic injury caused in this way by a traffic accident.

When the blunt force lands on the front of the chest, the left lobe of the liver is pushed towards the back. The falciform ligament fixes the liver to the diaphragm and prevents its movement, so that in blunt force trauma to the chest the attachment site on the liver bears the majority of the stress and is lacerated (4). When the blunt force is to the left of the falciform ligament, the lateral left lobe is pushed backwards and the laceration is to the left of the falciform ligament. When the blunt force is to the right of the falciform ligament, the laceration is also to the right of the falciform ligament (Fig. 3). The left hepatic vein and the left branches of the portal vein or hepatic bile duct may be ‘cut off’ in deep lacerations of this pattern.

#### Deceleration injury

**Figure 4.**
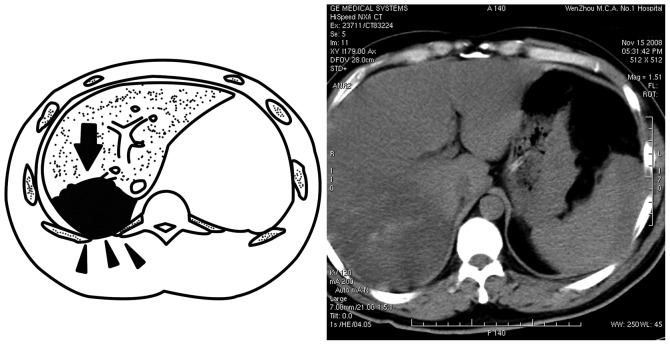
Deceleration injury of the right lobe of the liver. Propelled by huge forces and high speeds (black arrow), the liver moves towards and collides against the posterior abdominal wall (black arrowheads), lacerating this section of the liver. CT film shows a hepatic injury caused in this way by a traffic accident.

Another mechanism of liver injury in the right lobe of the liver is deceleration injury. This type of injury often occurs in traffic accidents and falls. When the rapidly moving body is suddenly stopped, the liver keeps moving and collides against the chest wall, resulting in a deceleration injury. The liver may also be propelled by huge forces at high speeds, then move towards and collide against the posterior abdominal wall, crushing or lacerating this section of the liver (Fig. 4).

#### Compression injury

**Figure 5.**
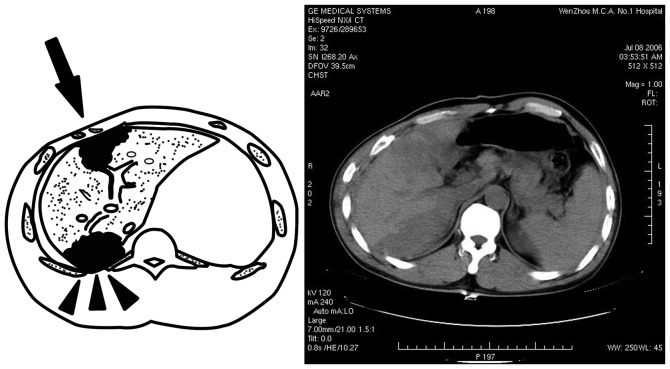
Compression injury of the right lobe of the liver. When the upper right quadrant is under compression in an anterior-posterior manner, the liver is crushed between the anterior and posterior walls of the rib cage (black arrow and black arrowheads, respectively) which lacerates the posterior and anterior sides of the liver at the same time. CT film shows a hepatic injury caused in this way by a traffic accident.

Compression injuries of the liver have previously been mentioned in other studies (1,2). The right lobe of the liver is relatively large and fixed so that when the upper right quadrant is under compression in an anterior-posterior manner, the liver has no room to escape and is crushed between the anterior and posterior walls of the rib cage. As a result, the posterior and anterior sides of the right lobe of the liver lacerate at the same time. Occasionally the laceration becomes a penetrating laceration or may even be completely destroyed if the compression force is large enough (Fig. 5).

#### Clinical implications

Although the present study identified three main patterns of blunt liver trauma, it also revealed that liver trauma is not decided by one single factor and that liver trauma in one patient is not confined to one single pattern. Certain superficial lacerations may be the result of the violent friction of the liver against the chest wall, particularly when the chest wall is broken and the surface is no longer smooth. When these lacerations are small, they are difficult to identify on CT scans. As the left lobe is smaller than the right, and is less fixed, in the present study it appeared that there were fewer compression and deceleration injuries in the left lobe than in the right lobe of the liver.

These findings indicate the importance of investigations into the circumstances in which the injury occurred. The forensic experts would be able to speculate the cause of the blunt liver trauma according to the patterns of the trauma when no other details were available (12). As for clinical doctors, attention should be paid to the speed at which a traffic accident occurs, the direction of the blunt force, the site the force hits and whether or not the victim was compressed, as these factors make a difference to the outcome of the injury. It is difficult to investigate the conditions under which the injuries take place, but careful and detailed investigation of the history is of great importance in the analysis of the trauma as the patterns and the severity of the liver injury are predominantly decided by the pattern of the blunt force.

Knowing that major hepatic trauma is always associated with thoracic injury (13), special attention should be paid to the chest; if possible, the chest should also be included in the CT scan. The CT scan provides the most valuable details, including site, type and severity of the trauma and whether there is also active bleeding (14–16). When performing CT scans and surgery, attention should be paid to the opposite side of the liver to which the blunt force landed. Occasionally the major injury is easily identified on the side where the forces land, but the injury on the opposite side is neglected.

Liver injuries caused by blunt force trauma are mainly associated with the right lobe. In the present study it appeared that the demarcation lines of the liver, be it on the surface or intrahepatically, were the sites where the trauma most often occurred. The anatomical demarcation line of the liver is the area where major hepatic veins are located. Severe injuries between Couinaud segments VII and VIII may injure the retrohepatic vena cava and the right hepatic vein. Injuries in the right lobe of the liver are extremely deep in the abdominal cavity and difficult to expose, which demands that surgeons select an appropriate incision to expose the laceration and avoid any improper movements of the liver that may make the laceration even worse. Occasionally this may lacerate the major hepatic vein or vena cava (4,17,18). The deep laceration along the falciform ligament caused by acceleration also demands great attention, as the left hepatic vein or the left branches of the portal vein or hepatic bile duct may be cut off by the injury.

#### Conclusion

Blunt liver trauma follows certain patterns and is mainly cause by three mechanisms: acceleration, deceleration and compression. The properties of the blunt force play a key role in blunt liver trauma. A thorough understanding of the mechanisms behind blunt liver trauma may aid doctors in the management of patients with this condition.
